# A phase 3, long-term, open-label safety study of Galcanezumab in patients with migraine

**DOI:** 10.1186/s12883-018-1193-2

**Published:** 2018-11-09

**Authors:** Angelo Camporeale, David Kudrow, Ryan Sides, Shufang Wang, Annelies Van Dycke, Katherine J. Selzler, Virginia L. Stauffer

**Affiliations:** 1 Eli Lilly Italia, Sesto Fiorentino, Italy; 2 California Medical Clinic for Headache, Santa Monica, CA USA; 3 UCLA David Geffen School of Medicine, Los Angeles, CA USA; 4 Eli Lilly and Company Corporate Center, Indianapolis, IN 46285 USA; 5 Neurology Department, AZ Sint-Jan Brugge, Brugge, Belgium

**Keywords:** Migraine, Headache, Galcanezumab, CGRP

## Abstract

**Background:**

Galcanezumab, a humanized monoclonal antibody that selectively binds to the calcitonin gene-related peptide, has demonstrated in previous Phase 2 and Phase 3 clinical studies (≤6-month of treatment) a reduction in the number of migraine headache days and improved patients’ functioning. This study evaluated the safety and tolerability, as well as the effectiveness of galcanezumab for up to 12 months of treatment in patients with migraine.

**Methods:**

Patients diagnosed with episodic or chronic migraine, 18 to 65 years old, that were not exposed previously to galcanezumab, were randomized to receive galcanezumab 120 mg or 240 mg, administered subcutaneously once monthly for a year. Safety and tolerability were evaluated by frequency of treatment-emergent adverse events (TEAEs), serious adverse events (SAEs), and adverse events (AEs) leading to study discontinuation. Laboratory values, vital signs, electrocardiograms, and suicidality were also analyzed. Additionally, overall change from baseline in the number of monthly migraine headache days, functioning, and disability were assessed.

**Results:**

One hundred thirty five patients were randomized to each galcanezumab dose group. The majority of patients were female (> 80%) and on average were 42 years old with 10.6 migraine headache days per month at baseline. 77.8% of the patients completed the open-label treatment phase, 3.7% of patients experienced an SAE, and 4.8% discontinued due to AEs. TEAEs with a frequency ≥ 10% of patients in either dose group were injection site pain, nasopharyngitis, upper respiratory tract infection, injection site reaction, back pain, and sinusitis. Laboratory values, vital signs, or electrocardiograms did not show anyclinically meaningful differences between galcanezumab dosesOverall mean reduction in monthly migraine headache days over 12 months for the galcanezumab dose groups were 5.6 (120 mg) and 6.5 (240 mg). Level of functioning was improved and headache-related disability was reduced in both dose groups.

**Conclusion:**

Twelve months of treatment with self-administered injections of galcanezumab was safe and associated with a reduction in the number of monthly migraine headache days. Safety and tolerability of the 2 galcanezumab dosing regimens were comparable.

**Trial registration:**

ClinicalTrials.gov as NCT02614287, posted November 15, 2015. These data were previously presented as a poster at the International Headache Congress 2017: PO-01-184, Late-Breaking Abstracts of the 2017 International Headache Congress. (2017). Cephalalgia, 37(1_suppl), 319–374.

## Background

In the 2015 Global Burden of Disease study, migraine was reported to be 1 of 8 chronic diseases affecting more than 10% of the world population [1], with higher prevalence among women (17%) than men (6%) [2]. Patients with migraine also have higher lifetime rates of depression, anxiety, panic disorder, sleep disturbances, chronic pain syndromes, musculoskeletal symptoms, ischemic stroke (migraine with aura), and suicide attempts [3–9]. Despite its prevalence, migraine continues to be underdiagnosed and undertreated.

Migraine-specific medications, such as triptans and ergotamines, as well as nonsteroidal anti-inflammatory drugs, are taken acutely to abort the migraine attack. However, for patients with frequent migraine attacks, and for whom abortive treatments are inadequately effective, preventive therapies are recommended [10–12]. It is estimated that approximately 39% of migraine patients would benefit from preventive pharmacotherapy to reduce the frequency of migraine attacks [2], which includes the ability to function at work and school, and interferes with family and social interactions [13].

For patients with chronic migraine, there are two preventive treatments considered as standard of care, onabotulinumtoxinA and topiramate, which are the most frequently prescribed medications for chronic migraine [14, 15]. In the US and Europe the use of beta blockers, calcium channel blockers, anticonvulsants, nonsteroidal anti-inflammatory drugs, and antidepressants as migraine preventive medications are proposed [10, 16, 17]. Although all of these medications are considered preventive treatment for episodic or chronic migraine, none of them were developed specifically to treat migraine, and some are not well tolerated [18].

During migraine attacks, serum concentrations of calcitonin gene-related peptide (CGRP) are significantly elevated in the external jugular vein [19, 20], implicating CGRP in the pathophysiology of migraine. Galcanezumab is a humanized monoclonal antibody that potently and selectively binds to CGRP without blocking the receptor, preventing CGRP-mediated biological effects [21]. In two 12-week Phase 2 [22, 23] and two 6-month Phase 3 [24] clinical studies of patients with episodic migraine, galcanezumab significantly reduced monthly migraine headache days (MHD) compared to placebo. The purpose of this study was to investigate the long-term safety, tolerability, and effectiveness of galcanezumab treatment in patients with migraine.

## Methods

This study was a Phase 3, multicenter, randomized, long-term, open-label study to assess the safety of two dosing regimens of galcanezumab, 120 mg/month (with initial loading dose of 240 mg) and 240 mg/month, for the treatment of episodic or chronic migraine. The study protocol was reviewed and approved by appropriate institutional review boards and was conducted according to Good Clinical Practice and the Declaration of Helsinki. Patients provided written informed consent before initiating study procedures. Enrollment began in December 2015 and the last patient completed the study (treatment phase and post-treatment phase) in September 2017. There were 28 clinical sites across 5 countries (United States, Canada, Hungary, Belgium, and France) that participated in the study.

### Patient selection

Eligibility for study enrollment was based on the results of migraine history, physical examination, neurological examination, clinical laboratory tests and electrocardiograms (ECGs). Key inclusion criteria were: 18–65 years of age; diagnosis of migraine as defined by the International Headache Society (IHS) International Classification of Headache Disorders (ICHD)-3 beta version [25] a history of at least 1 year of migraine headaches; migraine onset prior to age 50 years; prior to study entry, a history of 4 or more MHD per month on average for the past 3 months and a history of at least 1 headache-free day per month for the past 3 months. Key exclusion criteria were: prior exposure to galcanezumab (or any other CGRP antibody); use of any therapeutic antibody in the past 12 months; current treatment with preventive migraine medication; history of failure to respond to three or more classes of migraine preventive treatments (as defined by the American Academy of Neurology treatment guidelines Level A or Level B evidence [16]); presence of a medical condition that would preclude study participation, including pregnancy, presence of suicidal ideation within the past month, history of substance abuse or dependence in the past year, or recent history of acute cardiovascular events and/or serious cardiovascular risk based on history or ECG findings. Patients were allowed to take acute medications (except opiod and barbituates more than three times per month) for the treatment of migraine during the study, including triptans, ergots, nonsteroidal anti-inflammatory drugs and acetaminophen.

### Objectives

The primary objective was to evaluate the long-term safety and tolerability of galcanezumab (120 and 240 mg/month) for up to 1 year of treatment. Assessments included serious adverse events (SAEs), treatment-emergent adverse events (TEAEs), discontinuation rates, vital signs and weight, ECGs, laboratory measures, suicidal ideation and behavior using the Columbia Suicide Severity Rating Scale (C-SSRS) [26], and incidence of treatment-emergent anti-drug antibodies (TE-ADA).

Secondary objectives included the evaluation of efficacy measures to fully assess the longer-term effectiveness of galcanezumab in the prevention of migraine. The evaluation included overall change from baseline in the number of monthly MHD, headache days, responder analysis of ≥30%, ≥50%, ≥75, and 100% reduction in MHD, the percentage of patients who maintained a monthly MHD response, and change from baseline in the number of days acute treatment is taken for migraine or headache. Additional efficacy measures included patient-rated impression of illness improvement, change from baseline in functioning assessed by the Migraine-Specific Quality of Life questionnaire (MSQ) [27] and change from baseline in headache-related disability assessed by the Migraine Disability Assessment (MIDAS) scale [28, 29].

The number of MHD and headache days were reported by patients for the month prior to the study visit. Response rates were based on the reduction in number of MHD reported monthly and overall. Maintenance of response was a post-hoc assessment of patients meeting ≥50% response at any month and subsequently maintaining ≥40% response for at least two months or until the patient’s endpoint. This maintenance of response could range from ≥3 months to 12 consecutive months (including initial month of response).

### Clinical assessments

The C-SSRS evaluates the occurrence, severity, and frequency of suicide-related thoughts and behaviors during the assessment period. The scale includes suggested questions to solicit the type of information needed to determine if a suicide-related thought or behavior occurred [26].

The Patient Global Impression of Improvement (PGI-I) scale [30] is a patient-rated instrument that measures the improvement of the patient’s symptoms. It is a 7-point scale in which a score of 1 indicates that the patient is “very much better,” a score of 4 indicates that the patient has experienced “no change,” and a score of 7 indicates that the patient is “very much worse.”

The MSQ (v2.1) is a self-administered health status instrument that was developed to address physical and emotional limitations of specific concern to individuals suffering from migraine headaches. The instrument consists of 14 items that addresses 3 domains: (1) Role Function-Restrictive (RF-R), (2) Role Function-Preventive, and (3) Emotional Function [27]. The instrument was designed with a 4-week recall period and is considered reliable, valid, and sensitive to change in migraine [27, 31] with a 0 to 100 scale, with higher scores indicating a better health status.

The MIDAS was designed to quantify headache-related disability, recalled over a 3-month period. This instrument consists of five items that reflect the number of days reported as missing or with reduced productivity at work, home, or social events. The items are weighted in the final scores, with a higher value indicating greater disability [28, 29]. This instrument is considered highly reliable, valid, and is correlated with clinical judgment regarding the need for medical care [28, 29].

### Study design

The study was comprised of 3 study periods. Study Period 1 included initial screening procedures and washout of all migraine preventive treatments (3–45 days). In Study Period 2 (open-label treatment period), patients were randomized to treatment with one of two dosing regimens of galcanezumab (120 mg or 240 mg) that were administered subcutaneously once monthly for a total of 12 doses. Patients randomized to galcanezumab 120 mg received an initial loading dose of 240 mg (two injections of 120 mg each), and all subsequent doses were self- or caregiver-administered as a single injection of 120 mg monthly. Those randomized to galcanezumab 240 mg received two injections of 120 mg at each monthly dosing visit. Across the study, there were office visits at Months 1–3, 6, 9, and 12; Months 4, 5, 7, 8, 10, and 11 were telephone visits. Injections were delivered by prefilled syringe or by an investigational autoinjector. Each patient or caregiver received training on the use of the prefilled syringe and autoinjector. Patients were to keep track of their headaches, both migraine and non-migraine, experienced in the past 30 days, as well as the use of medication taken for the acute treatment of a migraine and non-migraine headache. Patients were required to report a migraine headache, headache or use of an acute medication for migraine or headache on a daily basis with a diary or log of their choice, and the daily log was reviewed at each monthly visit and documented in the case report form. Study Period 3 was a 4-month post-treatment period (washout phase), during which patients no longer received study medication, but continued to track headache information and received safety assessments. Patients who discontinued early from the treatment period could enter the post-treatment phase.

### Statistical analysis

Safety and effectiveness analyses were conducted on an intent-to-treat (ITT) basis, which included all randomized patients who received at least one dose of study drug. Change from baseline included only those patients who had a baseline and at least one post-baseline assessment.

Continuous variables without repeated measures were analyzed as change from baseline to the last observation carried forward (LOCF) endpoint. Continuous safety and efficacy variables with repeated measures were analyzed using mixed-model repeated measures (MMRM), which included the fixed categorical effects of treatment, treatment-by-visit interaction, visit, as well as the continuous fixed covariates of baseline and baseline-by-visit interaction. In addition, pooled investigative site was also included in the efficacy analyses.

Categorical variables with repeated measures were summarized and analyzed in a similar manner as mean changes by a categorical, pseudo likelihood-based repeated measures analysis using a generalized linear mixed model (GLIMMIX) procedure in SAS (SAS Enterprise Guide 7.1). Categorical variables without repeated measures were analyzed by Fisher’s exact test controlling for pooled investigative site.

The incidence of TE-ADA for each treatment group during the treatment period was summarized. Treatment-emergent ADA positive was defined as a ‘not present’ baseline ADA result and at least one ‘present’ post-baseline ADA result with a titer ≥1:20, or a ‘present’ baseline ADA result and a ‘present’ post-baseline ADA result with a ≥ 4-fold increase in titer (i.e., baseline titer of 1:10 increasing to ≥1:40 post-baseline).

All statistical tests were conducted at a 2-sided alpha level of 0.05. No adjustments for multiplicity were applied to any safety or effectiveness analyses.

## Results

**Fig. 1:**
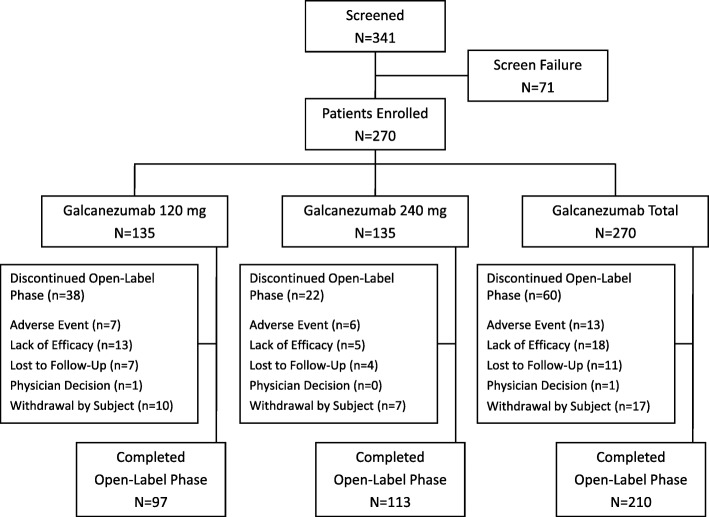
Patient cohort diagram through the treatment phase of the study

There were 341 patients screened for the study, of whom 270 patients enrolled. Overall completion rate for the treatment phase (Study Period 2) was 77.8% (*N* = 210) (Fig. 1) with a total of 60 patients (22.2%) who discontinued the treatment phase (Study Period 2). There were 236 patients (including some patients who discontinued treatment) who continued into the post-treatment phase (Study Period 3), and of these, 222 patients (94.1%) completed all 4 months.

Baseline demographics and clinical characteristics were similar between the dose groups, except for a statistically significant difference between dose groups in the mean number of MHD and age (Table 1). Patients enrolled in this study were 42 years of age on average, majority were female (83%) with a predominant diagnosis of episodic migraine (79%), and an average of 10.6 monthly MHD. Patients were diagnosed with migraine an average of 20.7 years prior to study enrollment, and a majority of patients (63%) reported prior use migraine preventive treatment, and 18.5% of the patients had one or more cardiovascular disease risk. The most common comorbid conditions (≥10%) were depression (16.7%), seasonal allergy (16.7%), drug hypersensitivity (15.6%), back pain (14.4%), insomnia (14.4%), anxiety (11.5%), and gastroesophageal reflux disease (10.4%). The mean MIDAS total score of 50% indicated very severe headache-related disability [32] and function was restricted, as indicated by the average MSQ RF-R score of 48.

**Table 1:** Demographics and Clinical Characteristics

	Galcanezumab 120 mg *N* = 135	Galcanezumab 240 mg *N* = 135
Age in years, mean (SD)	40.2 (11.7)	43.7 (11.0)*
Female, *n* (%)	110 (81.5)	113 (83.7)
Body mass index, kg/m^2^, mean (SD)	26.6 (5.4)	27.2 (5.8)
Race, *n* (%)		
Asian	2 (1.5)	0
Black	6 (4.4)	8 (5.9)
Multiple	23 (17.0)	19 (14.1)
White	103 (76.3)	108 (80.0)
Episodic migraine, *n* (%)	109 (80.7)	104 (77.0)
Cardiovascular Disease Risk Group, *n* (%)^a^	22 (17.1)	28 (19.9)
Comorbid conditions, mean (SD)^b^	4.3 (3.2)	4.7 (3.4)
Depression	19 (14.1)	26 (19.3)
Seasonal Allergy	24 (17.8)	21 (15.6)
Drug hypersensitivity	21 (15.6)	21 (15.6)
Back pain	18 (13.3)	21 (15.6)
Insomnia	19 (14.1)	20 (14.8)
Anxiety	15 (11.1)	16 (11.9)
Gastroesophageal reflux disease	12 (8.9)	16 (11.9)
Years since diagnosis, mean (SD)	20.2 (12.4)	21.3 (12.5)
Number of migraine headache days, mean (SD)	9.7 (5.8)	11.4 (6.7)*
Number of headache days, mean (SD)	5.0 (6.8)	6.1 (8.1)
Number of days with acute migraine medication use, mean (SD)	9.8 (6.6)	10.9 (7.2)
Prior preventive treatment, *n* (%)	81 (60.0)	88 (65.2)
Patient Global Impression - Severity, mean (SD)	4.7 (1.2)	4.7 (1.2)
Migraine Disability Assessment total, mean (SD)	45.8 (42.1)	54.0 (61.2)
Migraine-Specific Questionnaire Role Function-Restrictive domain score, mean (SD)	47.4 (19.2)	47.7 (18.4)

*SD* standard deviation

^a^Patients with a history or pre-existing condition listed in any of the following MedDRA Standardized Queries: Ischaemic Heart Disease, Hypertension, Cardiac Failure, Cardiomyopathy, Ischaemic CNS Vascular Conditions, Dyslipidaemia, Hyperglycaemia/New Onset Diabetes Mellitus

^b^Most common comorbid conditions (≥10%) are reported. *P < .05

The mean duration of exposure to galcanezumab was 318.5 days and 310.3 days in the 120 mg and 240 mg dose groups, respectively. Of the patients who discontinued the treatment period early, significantly more patients in the galcanezumab 120 mg dose group discontinued compared to the galcanezumab 240 mg dose group (*P* = .028). There were 4 patients who missed an injection at a home dosing visit, but they did complete the treatment phase, and the mean treatment compliance in this study was 95.8 and 96.9% in the galcanezumab 120 mg and 240 mg dose groups, respectively. There was no between-dose group difference in the percentage of patients who discontinued due to an adverse event (AE) (4.7% vs. 5.0% for galcanezumab 120 mg vs. 240 mg, respectively). In the galcanezumab 120 mg dose group, 2 patients discontinued due to injection site reaction, and 1 patient each discontinued due to injection site erythema, lethargy, migraine, and suicidal ideation. In the galcanezumab 240 mg dose group, 2 patients discontinued due to injection site reaction, and 1 patient each discontinued due to non-cardiac chest pain, paranoia, rash, tongue discomfort, and vertigo.

All of the 5 patients who discontinued due to an injection site-related TEAE had previous AEs at the injection site prior to discontinuation. Of these 5 patients, 4 patients discontinued after 6 or more self-administration dosing visits. One patient who had a severe injection site reaction discontinued after the tenth dosing visit due to progressive swelling around the site of the injection, with rash and pain that progressed from the previous injection that lasted a few days.

Ten patients reported SAEs, with 3 patients receiving galcanezumab 120 mg and 7 patients receiving galcanezumab 240 mg. Lumbar radiculopathy, migraine, and osteoarthritis occurred in the galcanezumab 120 mg dose group, while uterine leiomyoma embolization, cholecystitis, diverticulum intestinal, intervertebral disc protrusion, non-cardiac chest pain, pain in extremity and pneumonia occurred in the 240 mg dose group. The events of non-cardiac chest pain and migraine led to discontinuation. None of these events was reported by the study investigator to be associated with galcanezumab treatment.

Treatment-emergent AEs that occurred with ≥5% frequency in either dose group are summarized in Table 2. There were no significant differences between dose groups in the frequency of any of these events; however, there was a higher percentage of upper respiratory tract infection events in the galcanezumab 240 mg dose group (14.9%) compared with 120 mg group (7.0%). Most of the TEAEs were reported as mild-to-moderate in severity and there were no deaths. Across both dose groups, the most common (≥10% frequency) events were injection site pain, nasopharyngitis, upper respiratory tract infection, injection site reaction, back pain, and sinusitis. In addition, injection site bruising, injection site hematoma, injection site pruritus, and injection site induration were reported in > 2% in both galcanezumab dose groups combined. There were no SAEs related to injection sites.

**Table 2:** Treatment-emergent adverse events with a ≥ 5% frequency of occurrence in either galcanezumab dose group

Event	Galcanezumab 120 mg *N* = 129 *n* (%)	Galcanezumab 240 mg *N* = 141 *n* (%)
Patient with ≥1 TEAE	106 (82.2)	121 (85.8)
Injection site pain	22 (17.1)	28 (19.9)
Nasopharyngitis	23 (17.8)	18 (12.8)
Upper respiratory tract infection	9 (7.0)	21 (14.9)
Injection site reaction	15 (11.6)	13 (9.2)
Back pain	12 (9.3)	15 (10.6)
Sinusitis	14 (10.9)	13 (9.2)
Nausea	10 (7.8)	9 (6.4)
Injections site erythema	9 (7.0)	9 (6.4)
Arthralgia	8 (6.2)	8 (5.7)
Influenza	8 (6.2)	8 (5.7)
Dizziness	5 (3.9)	9 (6.4)
Injection site bruising	5 (3.9)	8 (5.7)
Myalgia	8 (6.2)	3 (2.1)
Weight increased	7 (5.4)	4 (2.8)

*TEAE* treatment-emergent adverse events

There were no statistically significant differences between dose groups in frequency of events

There were no clinically meaningful differences in laboratory parameters for either galcanezumab dose or between doses. No TEAE related to a laboratory analyte was reported as an SAE and none led to discontinuation. Elevated liver enzymes (as measured by alanine aminotransferase [ALT] or aspartate aminotransferase [AST] ≥3X upper limit of normal [ULN]; or alkaline phosphatase [ALP] ≥2X ULN; or total bilirubin level [TBL] ≥2X ULN at any time) were reported as TEAEs by 4 patients (galcanezumab 120 mg *N* = 3; galcanezumab 240 mg *N* = 1) and these elevations were not persistent.

Systolic blood pressure mean changes from baseline to each month ranged from − 1.45 to + 0.43 mmHg in the galcanezumab 120 mg group, and from − 1.65 to − 0.27 mmHg in the galcanezumab 240 mg group. Diastolic blood pressure mean changes from baseline to each month ranged from − 0.88 to + 0.87 mmHg in the galcanezumab 120 mg group, and from − 0.81 to + 0.23 mmHg in the galcanezumab 240 mg group. There were statistically significant, but not clinically important, mean increases from baseline in pulse at Months 1, 2, 3, and 9 that were of similar magnitude across both dose groups (range: 2.0 to 3.7 bpm; *P* < .01).

Few patients met criteria for treatment-emergent low systolic blood pressure, diastolic blood pressure, or pulse at any time (Table 3). There were no significant differences between galcanezumab dose groups in the frequencies of patients with treatment-emergent high systolic blood pressure or pulse at any time. There was a statistically significant increase in frequency of treatment-emergent high diastolic blood pressure in the galcanezumab 240 mg dose group compared to the 120 mg dose group (*P =* .046). Four patients had a sustained elevation in diastolic blood pressure (2 patients in each dose group), of whom 2 patients (1 in each dose group) had sustained elevation in systolic blood pressure. However, these were not sustained beyond 2 consecutive visits. A review of the patient-level data revealed that the increased blood pressure findings were transient, isolated events and likely represented normal variation in blood pressure. Three of these patients did have a TEAE of hypertension. Two patients with high diastolic blood pressure (1 in each dose group) also met the criteria for potentially clinically significant elevations at any time (Table 3).

**Table 3:** Treatment-emergent changes in blood pressure and pulse

Category	Galcanezumab 120 mg		Galcanezumab 240 mg	
	*N*	*n* (%)	*N*	*n* (%)
Elevated BP and pulse				
Sitting SBP ≥140 mmHg and ≥ 20 mmHg increase from baseline	120	5 (4.2)	124	4 (3.2)
Sitting DBP ≥90 mmHg and ≥ 10 mmHg increase from baseline	116	6 (5.2)	126	16 (12.7)*
Sitting pulse > 100 bpm and ≥ 15 bpm increase from baseline	129	3 (2.3)	139	5 (3.6)
Sustained elevation at 2 consecutive visits				
Sitting SBP	119	1 (0.8)	119	1 (0.8)
Sitting DBP	115	2 (1.7)	121	2 (1.7)
Sitting pulse	128	0	133	3 (2.3)
Potentially clinically significant elevation at anytime				
Sitting SBP ≥180 mmHg and ≥ 20 mmHg increase from baseline	129	0	139	0
Sitting DBP ≥105 mmHg and ≥ 15 mmHg increase from baseline	129	1 (0.8)	138	1 (0.7)

*BP* blood pressure, *DBP* diastolic blood pressure, *SBP* systolic blood pressure

*P < .05

Across the 12 months of treatment, the mean changes from baseline to LOCF endpoint in weight were small for both galcanezumab dose groups (≤1 kg). Thirteen patients in the galcanezuamb 120 mg dose group and 12 patients in the 240 mg dose group had treatment-emergent weight loss ≥7%; whereas, 17 patients in the 120 mg dose group and 21 patients in the 240 mg dose group had treatment-emergent weight gain ≥7%. Given that the observed categorical weight changes occurred in both directions (weight loss and weight gain), there does not appear to be a clear impact of galcanezumab on weight.

There was a statistically significant mean increase from baseline in temperature of 0.2^o^ F observed in each dose group at a single month (Month 1 for galcanezumab 120 mg [*P* < .01], Month 9 for galcanezumab 240 mg [*P* < .05]). A total of 10 patients overall experienced treatment emergent changes in body temperature. Five patients in the galcanezumab 120 mg dose group and 4 patients in the 240 mg dose group had low body temperatures (<96^o^ F and a decrease of ≥2^o^ F), and 1 patient in the 120 mg group had ≥101 °F and an increase of ≥2^o^ F. Since these changes were temporary and small, they were not considered clinically meaningful.

The percentage of patients with treatment-emergent abnormal changes from baseline in ECG measures were < 5% (Table 4). However, neither galcanezumab dose groups resulted in ECG changes or serious cardiovascular events of concern. There were no discontinuations due to treatment-emergent ECG findings.

**Table 4:** Change from baseline in electrocardiogram categorical measures

Category	Post Baseline	Galcanezumab 120 mg		Galcanezumab 240 mg	
		*N*	*n* (%)	*N*	*n* (%)
Heart rate	< 50 bpm and decrease ≥15	117	1 (0.85)	131	1 (0.76)
	> 100 bpm and increase ≥15	119	1 (0.84)	131	0
PR interval	< 120 msec	117	3 (2.56)	127	1 (0.79)
	≥ 220 msec	119	0	130	0
QRS interval	< 60 msec	120	0	131	0
	≥120 msec	118	0	131	1 (0.76)
QTcF	< 330 msec for males, < 340 msec for females	118	0	130	0
	> 450 msec for males; > 470 msec for females	118	2 (1.69)	130	1 (0.77)
	Potentially clinically significant:				
	> 500 msec	118	0	130	0
	Increase > 30 msec	118	2 (1.69)	130	4 (3.08)
	Increase > 60 msec	118	0	130	0

*bpm* beats per minute, *PR* pulse rate, *QTcF* QT interval adjusted for heart rate using Fridericia’s correction

Four patients experienced treatment-emergent suicidal ideation based on assessment with the C-SSRS. One of these patients (galcanezumab 120 mg dose) had a history of depression and was discontinued from the study after reporting suicidal ideation. The other 3 patients (galcanezumab 120 mg *N* = 2; 240 mg *N* = 1) had no prior lifetime history of suicidal ideation and continued in the study with no recurrence of suicidal ideation on the C-SSRS. None of the patients had emergence of suicidal behavior during treatment.

Anti-drug antibodies (ADA) were present at baseline in 8 (6.3%) out of 128 patients evaluable for TE-ADA in the galcanezumab 120 mg dose group, and in 12 out of 136 (8.8%) patients in the 240 mg dose group. Patients who developed TE-ADA included 16 (12.4%) patients in the 120 mg dose group and 10 (7.3%) patients in the 240 mg dose group. All of the patients who had TE-ADA also had neutralizing antibodies and the titers were generally low during this phase; the majority of the patients had maximum titers of 1:80 or below. Neutralizing ADA recognize the target-binding sites on galcanezumab and compete with binding to CGRP in vitro; an observable clinical effect requires sufficiently high titers of neutralizing ADA to effectively reduce the activity of galcanezumab in vivo.

Analysis of efficacy measures was a secondary objective in this study. Unless otherwise noted, the difference between galcanezumab 120 mg and 240 mg dose groups was not statistically significant on any efficacy measure.

**Fig. 2:**
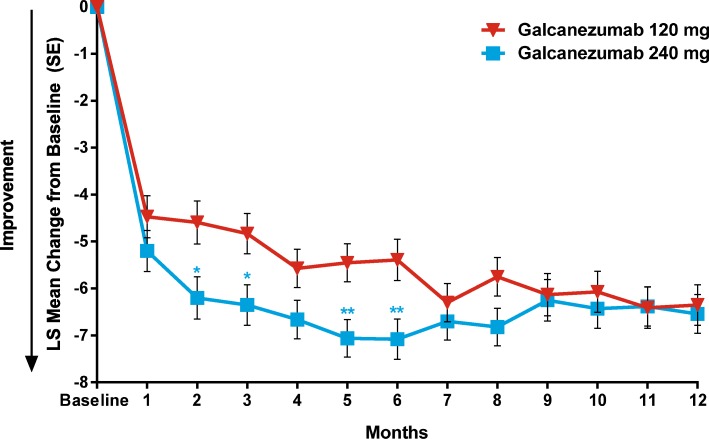
Overall mean change from baseline in the number of monthly migraine headache days. *P < .05; **P < .01. Overall least squares (LS) mean change from baseline in the number of migraine headache days for patients who were treated with monthly open-label injections of galcanezumab 120 mg or 240 mg

Compared to baseline, the overall reduction in the number of monthly MHD was 5.6 (95% CI: -6.3, − 5.0) and 6.5 (95% CI: -7.1, − 5.8) for patients treated with galcanezumab 120 mg and 240 mg, respectively (Table 5). Reduction in the mean monthly MHD was apparent as early as the first month and was sustained throughout the treatment period (Fig. 2).

**Table 5:** Overall change in monthly MHD, non-migraine headache days, and percentage reduction in monthly MHD

	Galcanezumab 120 mg *N* = 135	Galcanezumab 240 mg *N* = 135
Overall change from baseline in number of monthly MHD, mean (SD)	−5.6 (0.34)	−6.5 (0.33)
Overall change from baseline in monthly non-migraine headache days, mean (SD)	−2.2 (0.3)	−2.1 (0.3)
Overall change from baseline in number of days with acute medication use, mean (SD)	−5.1 (0.4)	−5.1 (0.4)
Percentage of patients who had ≥30% reduction in MHD	76.1%	80.9%
Percentage of patients who had ≥50% reduction in MHD	65.6%	73.7%
Percentage of patients who had ≥75% reduction in MHD	44.5%	52.5%
Percentage of patients who had 100% reduction in MHD	21.4%	21.8%

*MHD* migraine headache days, *SD* standard deviation

The overall mean reduction from baseline in the number of monthly non-migraine headache days averaged over 12 months was 2.2 and 2.1 in the galcanezumab 120 mg and 240 mg dose groups, respectively (Table 5).

In both galcanezumab dose groups, there were statistically significant within-group reductions from baseline in the number of monthly MHD or headaches with acute medication use at each month (*P* < .001). The overall mean reduction from baseline in number of monthly days with acute medication use for migraines or headaches was 5.1 in both dose groups (Table 5).

**Fig. 3:**
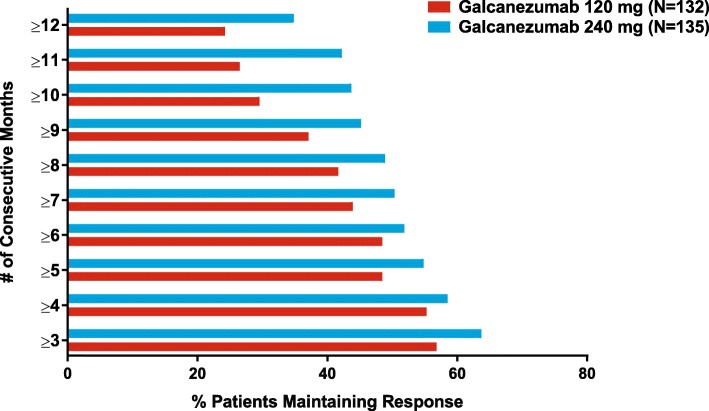
Maintenance of response. Percentage of patients treated with monthly injections of galcanezumab 120 mg or 240 mg, who had at least 50% or greater reduction from baseline in migraine headache days and maintained at least 40% reduction over 3 to 12 consecutive months

Response rate was defined as the mean percentage of patients meeting a pre-specified threshold in the reduction of the number of monthly MHD over Months 1 to 12. The overall response rates at each pre-specified threshold are summarized in Table 5. In each response category, there were more months where patients met that level of response in the galcanezumab 240 mg dose group compared to the galcanezumab 120 mg dose group. Of those patients who had at least a 50% reduction from baseline in the number of monthly MHD, the percentage who continued to maintain at least a 40% reduction over 3 to 12 consecutive months is shown in Fig. 3. In the galcanezumab 120 mg group, maintenance of response ranged from 48.5% (≥6 consecutive months) to 24.2% (up to 12 consecutive months), and in the 240 mg group, maintenance of response ranged from 51.9% (≥6 consecutive months) to 34.8% (up to 12 consecutive months).

Results from the Patient Global Impression of Improvement scale (PGI-I) is summarized in Table 6. In the galcanezumab 120 mg dose group 90 patients completed the PGI-I, and 80% of patients reported that they were “much or very much better” and 4% reported “no change” or “a little worse”. In the galcanezumab 240 mg dose group 112 patients completed the PGI-I, and 85% of patients reported that they were “much or very much better” and 8% reported “no change” or “a little worse”. There were no patients in either dose group who reported they were “much or very much worse”.

**Table 6:** Improvement in functioning and patient impression of illness improvement

	Galcanezumab 120 mg		Galcanezumab 240 mg	
	*N*	Mean (SE) or %	*N*	Mean (SE) or %
MSQ RF-R, mean increase (improvement)	130	31.6 (1.2)	135	33.4 (1.2)
MIDAS total, mean decrease (improvement)	124	−33.6 (2.1)	130	−32.7 (2.0)
Patient Global Impression – Illness Improvement at Month 12	90	–	112	–
Very much better		52.2%		52.7%
Much better		27.8%		32.1%
A little better		15.6%		7.1%
No change		3.3%		7.1%
A little worse		1.1%		0.9%
Much worse		0%		0%
Very much worse		0%		0%

*MIDAS* Migraine Disability Assessment, *MSQ RF-R* Migraine-Specific Questionnaire Role Function – Restrictive, *SE* standard error

Patients in both galcanezumab dose groups had improved functioning, as assessed by the MSQ RF-R domain, with increases from baseline in least squares (LS) mean scores of 31.6 and 33.4 for the 120 mg and 240 mg dose groups, respectively. Additionally, both galcanezumab dose groups had reduced headache-related disability, as assessed by the MIDAS total score, with LS mean reductions from baseline of − 33.6 and − 32.7 for the 120 mg and 240 mg dose groups, respectively.

## Discussion

In this 12-month open-label study of once monthly subcutaneous injections of galcanezumab 120 mg and 240 mg as a preventive treatment for migraine, the safety and effectiveness profile observed was consistent with previous studies: two Phase 2 studies [22, 23], and two Phase 3 studies in patients with episodic migraine [24], and one Phase 3 study in patients with chronic migraine [33].

Tolerability to galcanezumab was demonstrated by the overall high study completion rate, which was 77.8% through all 12 months of treatment. In patients who completed the study, treatment compliance was > 95% and included at least half of the study visits being self-administered injections at home. Furthermore, the percentage of discontinuations due to AEs was low (< 5% combined doses), and few SAEs occurred (< 4% combined doses, and none considered related to treatment). This is in contrast to long-term treatment with topiramate, which is currently the most prescribed preventive migraine medication, which showed higher rates of study discontinuation and discontinuation due to adverse events [34, 35].

In this study, where patients or caregivers administered subcutaneous injections of galcanezumab, AEs of particular interest were those associated with the injection site. Approximately one-third of the patients experienced an injection site AE, the reason for which 5 patients discontinued. Most of the TEAEs related to injection sites were mild or moderate in severity and occurred on the day of injection, and the majority were resolved by the next day. Of the 5 patients who discontinued due to an injection site AE, 4 did so after multiple self-administrations. None of the TEAEs appeared to be different between the doses with the exception of the reported AE of upper respiratory tract infection. However, the cluster of events under upper respiratory infections show a similar incidence between the galcanezumab 120 mg dose group (35.7%) and the 240 mg dose group (37.6%). In addition, safety data from the Phase 3, double-blind, placebo-controlled studies for all three treatment groups (galcanezumab 120 mg, galcanezumab 240 mg, and placebo) showed a similar incidence of the AE of the upper respiratory tract infection [36].

The safety of galcanezumab was supported by generally temporary and minimal changes from baseline in laboratory values, vital signs, ECG parameters, and weight. There were no clinically meaningful differences in laboratory parameters between the galcanezumab doses, based on mean changes from baseline to endpoint, as well as treatment-emergent changes (i.e., treatment-emergent abnormal, low, or high). These findings are supported by safety analyses performed with data pooled from two 6-month and one 3-month, Phase 3, double-blind, placebo-controlled studies [36].

Migraine may be associated with increased risk of suicidal ideation or behavior as reported by several studies [8, 9, 37]. In the current study, nearly 17% of the patients had comorbid depression, but treatment-emergent suicidal behavior was not reported. Four patients reported suicidal ideation as assessed by the C-SSRS. Three of these patients did not have a history of depression, but had a one-time incidence of treatment-emergent suicidal ideation as assessed by the C-SSRS, and all 3 patients continued in the study. One patient discontinued from treatment due to treatment-emergent suicidal ideation.

Immunogenicity is an important topic in therapies using monoclonal antibodies. Of particular interest is the development of ADA and their relevance in contributing to possible allergic drug reactions, neutralization of therapy (possibly reducing efficacy), and potential association with AEs. In this study, there were 26 patients who had TE-ADAs. Of these, only four patients reported one or more hypersensitivity events (specifically, rash and puritis) during the treatment phase and these events were mild-to-moderate in severity and all were resolved by the end of the treatment phase. Future analyses based on integrated safety and efficacy summaries across galcanezumab studies will allow for larger samples sizes, and potentially provide a better understanding of immunogenicity.

Effectiveness of treatment with galcanezumab was demonstrated by both doses on multiple migraine-relevant outcome measures over 12 months of treatment including: reduction in the number of monthly MHD; reduction in the number of days having a non-migraine headache; response rates; maintenance of response; and reduction in the frequency of acute medication use. The findings for the reduction in the number of monthly MHDs and response rates at the 50, 75, and 100% are consistent with findings reported by Ashina et al. 2017 in a 1-year open-label extension study of erenumab, a monoclonal antibody that blocks the CGRP receptor [38]. In addition, over 80% of the patients reported a disease improvement as measured by PGI-I to be “much better” or “very much better”. Also, functioning was greatly improved, with changes from baseline in MSQ RF-R scores being three-fold greater than the within-group minimally important difference of 10.9 that has been determined for this domain [39]. In addition, headache-related disability was reduced from very severe to moderate.

This study is limited by the relatively small sample size, which precludes detection of any rare AE that may occur with long-term galcanezumab treatment. Patients with recent or serious cardiovascular conditions were excluded from participating in galcanezumab clinical studies, therefore caution should be used when treating these patients. In addition, there are limited data from the use of galcanezumab in pregnant women as they were excluded from participating in the galcanezumab studies. Interpretation of the effectiveness outcomes is limited by the open-label study design without comparison to placebo or another active treatment, and while the daily diaries collected the same information (migraine headache, headache, or use of acute medications), the use of a paper diary is a limitation of the study since an electronic diary can provide monitoring of daily entry and minimize recall bias. Nevertheless, the effectiveness results are similar to those of the more rigidly controlled Phase 3 studies. Lastly, in this study, the majority of the patients met criteria for episodic migraine and further assessment of the patients with episodic compared to chronic migraine will be explored in a future publication.

## Conclusion

In summary, there were no new safety findings identified during 12 months of treatment with galcanezumab; favorable tolerability was evidenced by low discontinuation rates due to AEs, and TEAEs were transient and predominantly rated as mild or moderate in severity. Furthermore, there were no meaningful differences between galcanezumab doses with respect to measures of safety and tolerability. Although the study design was uncontrolled and open-label, the totality of migraine headache reduction along with improvement in functioning and disability, are considered to be clinically meaningful [39]. Results from this study confirm the long-term effectiveness of galcanezumab in patients with migraine.

## Abbreviations

AEs: Adverse events
ALP: Alkaline phosphatase
ALT: Alanine aminotransferase
AST: Aspartate aminotransferase
BP: Blood pressure
bpm: Beats per minute
CGRP: Calcitonin gene-related peptide
C-SSRS: Columbia Suicide Severity Rating Scale
DBP: Diastolic blood pressure
ECGs: Electrocardiograms
GLIMMIX: Generalized linear mixed model
ICHD: International Classification of Headache Disorders
IHS: International Headache Society
ITT: Intent-to-treat
LOCF: Last observation carried forward
LS: Least squares
MHD: Migraine headache days
MIDAS: Migraine Disability Assessment
MMRM: Mixed-model repeated measures
MSQ RF-R: Migraine-Specific Quality of Life questionnaire Role Function – Restrictive
PGI-I: Patient Global Impression of Improvement
PR: Pulse rate
QTcF: QT interval adjusted for heart rate using Fridericia’s correction
SAEs: Serious adverse events
SBP: Systolic blood pressure
SD: Standard deviation
SE: Standard error
TBL: Total bilirubin level
TE-ADA: Treatment-emergent anti-drug antibodies
TEAEs: Treatment-emergent adverse events
ULN: Upper limit of normal
